# Climate Change—A Global Threat Resulting in Increasing Mycotoxin Occurrence

**DOI:** 10.3390/foods12142704

**Published:** 2023-07-14

**Authors:** Jovana Kos, Mislav Anić, Bojana Radić, Manuela Zadravec, Elizabet Janić Hajnal, Jelka Pleadin

**Affiliations:** 1 Institute of Food Technology, University of Novi Sad, Bulevar Cara Lazara 1, 21000 Novi Sad, Serbia; jovana.kos@fins.uns.ac.rs (J.K.); bojana.radic@fins.uns.ac.rs (B.R.); elizabet.janich@fins.uns.ac.rs (E.J.H.); 2 Croatian Meteorological and Hydrological Service, Ravnice 48, 10000 Zagreb, Croatia; mislav.anic@cirus.dhz.hr; 3 Department of Veterinary Public Health, Croatian Veterinary Institute, Savska Cesta 143, 10000 Zagreb, Croatia; zadravec@veinst.hr

**Keywords:** mycotoxins, moulds, environmental conditions, global warming, geographic regions, prevention, prediction

## Abstract

During the last decade, scientists have given increasingly frequent warnings about global warming, linking it to mycotoxin-producing moulds in various geographical regions across the world. In the future, more pronounced climate change could alter host resilience and host–pathogen interaction and have a significant impact on the development of toxicogenic moulds and the production of their secondary metabolites, known as mycotoxins. The current climate attracts attention and calls for novel diagnostic tools and notions about the biological features of agricultural cultivars and toxicogenic moulds. Since European climate environments offer steadily rising opportunities for *Aspergillus flavus* growth, an increased risk of cereal contamination with highly toxic aflatoxins shall be witnessed in the future. On top of that, the profile (representation) of certain mycotoxigenic *Fusarium* species is changing ever more substantially, while the rise in frequency of *Fusarium graminearum* contamination, as a species which is able to produce several toxic mycotoxins, seen in northern and central Europe, is becoming a major concern. In the following paper, a high-quality approach to a preventative strategy is tailored to put a stop to the toxicogenic mould- and mycotoxin-induced contamination of foods and feeds in the foreseeable future.

## 1. Introduction

During the production and storage of raw materials and final food and feed products, moulds frequently appear as contaminants capable of producing a broad spectrum of secondary metabolites, the most significant of which are mycotoxins. Mycotoxins are a large and diverse group of naturally occurring fungal toxins, many of which have been implicated as chemical agents of toxic disease in both humans and animals. The major genera of mycotoxigenic moulds are *Aspergillus*, *Fusarium*, and *Penicillium*. Among the hundreds of mycotoxins uncovered so far, special attention has been paid to aflatoxins and ochratoxins, especially aflatoxin B1 (AFB1) and ochratoxin A (OTA), as well as *Fusarium* mycotoxins fumonisins (FUMs), zearalenone (ZEN), deoxynivalenol (DON), and T-2/HT-2 toxin (T-2/HT-2), given that all of the aforementioned have been detected across the globe. Mycotoxins are known to cause various toxic effects in both humans and animals, including hepatotoxicity, nephrotoxicity, neurotoxicity, mutagenicity, carcinogenicity, and immunosuppression [1,2,3]. 

Among them, the most toxic AFB1 is a frequent contaminant of various commodities, primarily cereals, nuts, and spices, and represents a highly toxic mycotoxin [4,5,6,7,8,9]. OTA is the most prominent and the most toxic representative of the ochratoxin group, and contaminates a wide range of foodstuffs, including cereal-based products, spices, tree nuts, coffee, dried fruits, and wine [10,11]. *Fusarium* mycotoxins, FUMs, ZEN, DON, and T-2/HT-2 toxins mainly contaminate cereals such as maize, wheat, rice, oats, and barley [12]. Nevertheless, the spectrum of mycotoxins of increasing concern in food and feed is rather broader, and therefore masked mycotoxins, emerging mycotoxins, and modified forms of certain mycotoxins are of increased global concern.

It has been established that the level of colonisation of a given foodstuff or feedstuff with toxicogenic moulds depends on the interaction of numerous factors, such as the type of substrate and nutrients’ availability, the percentage of substrate and environmental humidity, the maturity of the colony, the presence of other moulds, competition with other microorganisms, insect-inflicted substrate damage, and so forth [13,14]. Crop cultivation procedures and the current climate also influence the occurrence of various harmful agents, primarily insects, which by damaging crops can also cause contamination with toxicogenic moulds and mycotoxins. Mycotoxin production is greatly influenced by dominating environmental conditions; that is, climate features typical of various geographic regions, whose features vary on an annual basis [8,15,16,17].

Research has shown that the effects of climate change, such as elevated carbon dioxide levels, environmental temperature rise, and the interchange of extreme droughts with extreme rainfalls, have a significant impact on mould growth and mycotoxin occurrence [15,18]. Long-term changes in environmental temperature, humidity, precipitation amount, and weather extremes’ frequency already influence agricultural practices, production, and quality of food crops; consequently, these combined effects could induce decreased agricultural yields (by 10–30% in many regions). Given that the latest scientific notions point towards ever more substantial climate change, it is to be expected that in the foreseeable future the latter change shall have a significant impact on toxicogenic moulds’ occurrence, and hence the prevalence, frequency of occurrence, and predominating types of mycotoxins in various geographical regions around the globe, posing a major health and economic risk and global food safety threat [19].

Climate-change-related issues are expected to become a major 21st century environmental concern, whose effects are mirrored in changes in precipitation amounts, intensified and more frequent weather extremes, sea level rise, downsizing of potable water stocks, desert area enlargement, more pronounced disease outbreak threats, and extinction of numerous biological species. From a geographical standpoint, the highest risk of climate-change-induced mycotoxin contamination is foreseen in developed countries with moderate climates. Higher environmental temperatures combined with extreme precipitation amounts or prolonged droughts increase the level of stress suffered by plants, making all cereals, especially maize, even more prone to mould infection and mycotoxin contamination. As compared to the past, in which *Fusarium* (*F*.) and *Aspergillus* (*A*.) mycotoxins were more frequently seen in southern Europe, *Fusarium* mycotoxins will shift to northern Europe, while *Aspergillus* species will primarily be seen in south and central Europe [6,7,8,20,21]. The European Food Safety Authority (EFSA) predicts that the effects of climate change on the occurrence of mycotoxins will be regional and mostly detrimental, but possibly also beneficial for a particular geographical area [19]. However, the impact of climate change on the formation of mycotoxins is difficult to predict due to the complex interplay of various factors [22].

This article provides an overview of the scientific notions gathered insofar and the impact of climate change on mycotoxin occurrence anticipated in certain geographic regions around the globe. It aims to emphasize the significance of preventative strategies employed to the end of reducing climate impact on an already increased contamination with toxicogenic moulds and mycotoxins; both of the above are recognized as major food and feed contaminants.

## 2. Factors Affecting Climate Change

The enhanced exploitation of fossil fuels since the beginning of the industrial revolution is considered the main driver of climate change on Earth. Continuously growing atmospheric concentrations of the three main greenhouse gases (carbon dioxide, CO_2_; methane, CH_4_; and nitrous oxide, N_2_O) reached the highest values in 2021 [23]. The most evident consequence of the increase in the concentration of greenhouse gases is the increase in atmospheric temperature. The global 10-year average for the decade 2011–2020 is estimated to be 1.09 °C above the 1850–1900 pre-industrial average [24]. Global warming has altered distribution and area suitability for numerous species, as well as the timing of key phenological events. Observations are showing an earlier onset of leaf unfolding and flowering during spring, as well as a delay in leaf colouring and fall in autumn [25]. 

Furthermore, all components of the global water cycle have been altered due to climate change. Rising global air temperatures have increased atmospheric evaporative demand, which has enhanced moisture loss from evapotranspiration [26]. Observations from weather stations and satellites indicate shifts in precipitation patterns. The incidence and severity of drought events have increased in many parts of the world. On the other hand, the incidence of extreme rainfall and associated increases in the frequency and magnitude of river floods have been observed [24]. Despite mould’s general resilience to elevated carbon dioxide concentrations, when working in combination with other factors, such as stress, droughts, insect-inflicted plant damage, and crop phenology changes (for instance, changes in crop blooming and maturation time), this gas may have a substantial indirect impact on mycotoxin production [27,28]. Climate change has affected the various regions of the world differently, and an overview is summarized in Table 1. The most widely cited estimation of global mycotoxin occurrence by the Food and Agriculture Organization (FAO) indicated that 25% of global agricultural commodities are contaminated with mycotoxins [1]. However, increasingly recent studies indicate that the percentage of contaminated agricultural commodities is significantly higher, and very often exceeds 80% [29,30,31,32]. The recently increased occurrence of mycotoxins, as well as newly identified mycotoxins, is more often explained by a combination of the improved sensitivity of analytical methods and the impact of climate change [32,33]. The application of new-generation analytical methods enables the detection of mycotoxins at significantly lower concentrations than in the past, which directly affects the increase in the percentage of contaminated samples. Further, many scientific studies have confirmed the influence of climate change on the increases and differences in mycotoxin occurrence and level of contamination. 

Scientists across the globe are increasingly investigating the relationship between climate change on mycotoxin occurrence in different commodities. Studies have shown that environmental stress has a significant bearing on mould-induced mycotoxin production [34,35,36]. Increased levels of food and feed contamination may come as a result of contamination with mycotoxins currently typical of the region, or as a consequence of “relocation” of certain mycotoxins from their previous domiciles to novel geographic regions in which they have never been documented before, at least not in significant levels. Climate-change-induced stress may result in the nascence of novel mycotoxins never seen or recognised before, capable of severely reducing food and feed availability, particularly in developing countries [37]. According to Paterson and Lima [38], the basic premise of the effect of climate change on mycotoxins is the migration of thermotolerant moulds (e.g., *A. flavus*) from tropical regions to regions with currently temperate climates.

The European Food Safety Authority foresees the effects of climate change on mycotoxin prevalence to be regional and mostly harmful, but possibly also beneficial for certain geographic regions [19,20]. It is to be expected that climate change will also yield global change in the prevalence and geographic distribution of insect populations [13,39,40], known for their significant impact on toxigenic mould-induced crop infections and hence more severe crop contamination. Studies have shown that climate change may be responsible for one-third of the global food yield variability (the latter variability referring to the essential and vital foodstuffs) and has a negative impact on the nutritive value of the foodstuffs in question [19,41]. 

## 3. Conditions Facilitating Mycotoxin Production

**Figure 1:**
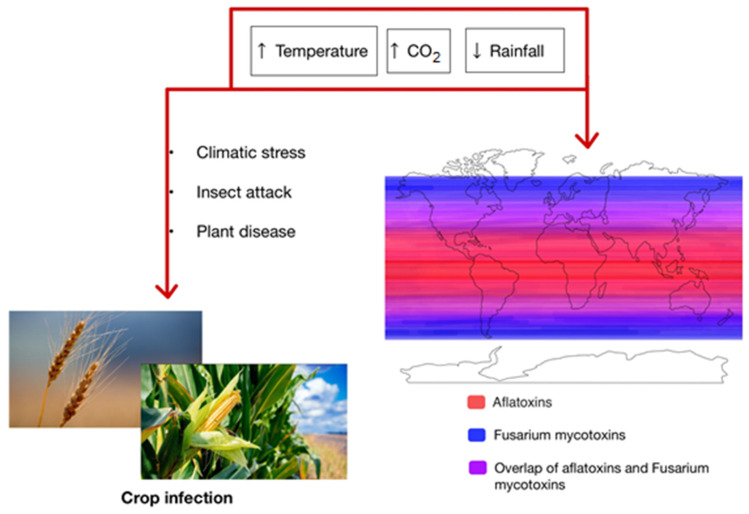
Influence of different factors on the occurrence of mycotoxins worldwide.

It is known that key factors influencing mould growth and mycotoxin production are environmental temperature, water activity (a_w_), and pH value (Table 2). The influence of different factors on the occurrence of mycotoxins worldwide is shown in Figure 1.

### 3.1. Aflatoxins

It was evidenced that aflatoxins primarily produced by the *Aspergillus* species are expected to be found in higher concentrations in tropical and subtropical areas [42,43]. In warm and humid subtropical and tropical climates, various cereals, especially maize, represent ideal milieus for the colonization with predomination of *A. flavus* and *A. parasiticus* species. Environments optimal for aflatoxin production are those with a temperature of 33 °C and a_w_ of 0.99 [44].

### 3.2. Ochratoxin A

OTA is produced by several *Aspergillus* moulds, primarily *A. ochraceus*, *A. niger*, and *A. carbonarius*, as well as by the *Penicillium* species, mainly *P. verrucosum* [45,46]. *A. ochraceus* has been found in a vast number of foodstuffs, mainly in cereals, beans, spices, dry fruits, tree nuts, and seeds [47]. The temperature range favouring *A. ochraceus* growth is much wider than that favouring OTA production (8–37 °C) [48]. The largest amounts of this mycotoxin are produced at a_w_ 0.98 regardless of the temperature, while, dependent on the isolate, the most productive temperatures are those of 25–30 °C. The type of food, that is, its nutritive composition and status, is of the utmost importance for OTA production [25]. Mitchell et al. [49] and Batillani et al. [50] proved that the temperature optimal for *A. carbonarius* and *A. niger* growth is 35 °C, while the mould ceases to grow at temperatures lower than 15 °C and higher than 45 °C, the optimal a_w_ thereby being 0.93–0.99. However, data on the influence of environmental conditions on *Aspergillus*-generated OTA production are scarce. *P. verrucosum* efficiently colonises various crops, especially those growing in humid and colder climates around the globe, preferring the colonisation of insufficiently dried wheat and barley. *P. verrucosum* can grow within a broad temperature range (0–35 °C). Even though the temperature optimal for OTA growth in cereal grains is 25 °C, during prolonged storage the toxin can appear even at temperatures of 5–10 °C, provided that the storage environment is sufficiently humid. Dependent on the storage duration, the optimal a_w_ spans from 0.90 to 0.95, with the required minimum aw of 0.83–0.85 [51].

### 3.3. Fusarium Mycotoxins

*Fusarium* mycotoxins are more likely to be found in regions characterized by moderate climates, while heavy rains favour *Fusarium* mycotoxins’ production [16,52]. Research completed many years ago established that the optimal growth of *Fusarium* moulds *F. culmorum*, *F. poae*, *F. avenaceum*, and *F. tricinctum* calls for the temperatures of 20–25 °C, the lower limit being 5–10 °C and the upper limit being 35 °C [53]. The *Fusarium* moulds listed above synthesize numerous *Fusarium* mycotoxins, most of them contaminating cereals [54,55,56]. Optimal mould growth (within the optimal temperature frame) for a_w_ is considered to be in the range of 0.98–0.995, the minimum thereby being 0.90–0.91 [57]. 

The optimal temperature for *F. graminearum*- and *F. culmorum*-generated DON production is between 25 and 30 °C, depending on the incubation length [58]; however, to maintain toxin production, the temperature should not drop below 11 °C. DON production generally requires a much narrower a_w_ and temperature range as compared to the *Fusarium* moulds’ growth. The available data on ecological profiles of zearalenone production are scarcer [44]. Most of the *F. verticillioides* isolates primarily produce FUM B1 and smaller amounts of FUM B2, B3, and B4. The temperature range facilitating the growth of the above mould is far wider than the range needed for the production of FUMs listed above. The mould in question grows at temperatures of 4–37 °C, the optimum thereby approximating 30 °C, while FUMs are mostly produced within the temperature range of 10–37 °C, the optimum thereby being 15–30 °C [59,60].

ZEN, in addition to the previously mentioned mycotoxins, is one more representative of regulated mycotoxins. It is produced by numerous species of *Fusarium* moulds, including *F. graminearum*, *F. roseum*, *F. tricinctum*, *F. sporotrichioides*, etc. The optimal temperature for these moulds and ZEN synthesis is 25 °C, at a_w_ in the range of 0.90–0.995. ZEN is the most commonly detected as a contaminant of maize, wheat, barley, oats, and sorghum in temperate and humid conditions [61,62].

**Table 2:** Mycotoxin-producing mould species and their abiotical optimal growth conditions [61].

Mycotoxin	Mould	Temperature Range (°C)	Optimal Temperature (°C)	Water Activity (a_w_)	pH
AFs	*A. flavus* *A. parasiticus*	10–48 12–42	33 32	0.80–0.99 0.80–0.99	2–10 3–8
OTA	*A. ochraceus* *P. verrucosum* *A. niger*	10–40 0–31 6–47	37 20 36	0.80 0.86 0.77–0.92	3–10 6–7 2–6.5
FUM	*F. verticilloides* *F. proliferatum*	2.5–37 5–37	25	0.90–0.99	2.4–3
ZEN	*F. culmorum*	0–31	21	0.96	3–9
DON	*F. graminearum*	5–37	25	0.99	2.4–3

## 4. Climate Change and Effect on Mycotoxin Occurrence in Europe

Europe is warming even faster than other continents, at an average rate of about 0.5 °C per decade during the period 1991–2021 [23,63]. The incidence of warm days, nights, and heatwaves over Europe has increased, while the incidence of cold spells has decreased. Mean precipitation has slightly increased during recent decades in Northern, Western, Central, and Eastern Europe. The incidence of extreme rainfall has increased in Northern and Eastern Europe. The Mediterranean region is experiencing more frequent and severe droughts and becoming more arid [24]. 

In terms of mycotoxins, Europe has a high awareness of their prevention and control, as well as the most rigorous, extensive, and detailed regulations on mycotoxins in food and feed in the world. However, mycotoxin contamination in different foodstuffs and feedstuffs is a growing concern. Every year, crops across Europe are seriously contaminated with mycotoxins influenced by the changing weather, and their use and processing are therefore restricted, resulting in huge economic losses. Especially in years with extreme weather conditions, crops are more susceptible to mould contamination, and the content of mycotoxins in food is significantly increased, which is more likely to pose a threat to food safety [64]. 

Among the crops, maize and wheat, as the most commonly used and most important cereals in Europe, are the most susceptible crops to mycotoxin contamination, especially when extreme weather conditions are recorded. The influence of weather conditions on the appearance of mycotoxins in maize and wheat has been investigated in many scientific papers. 

In Europe, the maize-growing season usually lasts during spring and summer, while the wheat-growing season lasts longer, usually from the second part of autumn to the first part of summer. In both maize and wheat, higher precipitation during the second part of spring influences favourable conditions for *Fusarium* species that result in increases of certain mycotoxins: the highest being DON, followed by ZEN and FUMs (in maize). On the other hand, a lack of precipitation and higher air temperatures during the summer months are more favourable for *Aspergillus* species and therefore more frequent maize contamination with aflatoxins. In some parts of Europe, especially in southern Europe, extremely hot summers have already led to changes in crop cultivation ecosystems. All the effects mentioned represent a great concern, since maize and wheat represent staple food and feed ingredients and their contamination is a further problem for the whole food and feed chain [6,8,65,66].

### 4.1. Aspergillus Mycotoxins

It is believed that temperature and humidity rises as a result of climate change are most probably responsible for the increased *Aspergillus* mycotoxins contamination witnessed in southern Europe from the early 2000s to the present; at the same time, a steady increase in contamination occurrence has been seen in northern Europe as well. Among *Aspergillus* mycotoxins, in previous decades, the most frequently detected were AFB1 in maize and AFM1 in milk. Reports from many European countries have indicated increased frequency of aflatoxins [67,68].

For example, research conducted in two European countries, Croatia and Serbia, represents one of the most extensive investigations into the implications of climate change on the occurrence of aflatoxins, including data from a period of thirteen years [8]. This study showed vast maize contamination during 2013 due to the climate impact seen across the 2012 maize cultivation season, the climate at the time practically mimicking tropical and subtropical conditions [4,6,8,69]. In these countries, the weather conditions, characterized by high air temperatures and a lack of precipitation, were again recorded during the summer months, which resulted in repeated aflatoxins contamination of maize during the several following years. 

In Serbia, extremely hot summers have already resulted in changes in maize-growing ecosystems, which have led to a more frequent occurrence of *A. flavus* and contamination with AFB1 [6,7,70]. Significant contamination of maize with aflatoxins was repeated in several years (2012, 2013, 2015, 2017, 2021), which consequently led to the contamination of milk and dairy products with aflatoxin M1 [14,65]. During those years, in the summer months, lack of precipitation was followed by temperatures that were very often above the annual average (>35 °C). According to the report of the Republic Hydrometeorological Service of Serbia, 13 out of the 15 hottest years in the Serbian meteorological past were registered after 2000 [70,71]. 

Assunção et al. [72] gave an overview of climate change and the health impact of aflatoxin exposure in Portugal. They indicated that the consumption of milk and maize-derived products, especially breakfast cereals, represents the greatest risk of aflatoxin exposure to the Portuguese population. Further, the authors also identified maize as the matrix that is the most susceptible to aflatoxin contamination in a climate change scenario. They predicted that, besides in Portugal, contamination of AFB1 in cereal crops will become a food safety issue in Europe within the next 100 years.

One of the most extensive, recently published, scientific opinions on the risk assessment of aflatoxins in food based on the results from more than 25 European countries was published by the EFSA. This report indicates that climate change is anticipated to increase the level of aflatoxin contamination from low to medium in food in Europe, especially in maize in countries in which maize cultivation is common (e.g., France, Italy, and Romania). Further, the EFSA report also highlighted the relationship between the contamination of maize with AFB1 and subsequent contamination of milk and dairy products with AFM1. In conclusion, this scientific opinion highlighted the importance of continuous monitoring of aflatoxin occurrence, since potential increases are expected due to more frequent weather extremes and climate change [73].

Besides these reports, there is a constantly growing number of studies that indicate an increased trend of aflatoxin risk in Europe due to climate change [9,10,67,68,70].

### 4.2. Fusarium Mycotoxins

In recent years, an upward trend has been observed in the incidence of certain *Fusarium* mycotoxins in different agricultural commodities, especially in maize, across European countries. This trend is likely associated with the change in climatic conditions and the agronomic practices used [74].

Results from many European countries indicate that the most frequently occurring *Fusarium* mycotoxin is DON [64,75]. Luo et al. [64] summarized results for DON occurrence in different cereals, from 15 European countries from 2010 to 2019, and concluded that among regulated mycotoxins DON had the most serious contamination and the widest distribution. Depending on the cereals, DON is detected at different contamination frequencies that are usually highest in wheat and maize, while the range of the determined concentration mainly depends on weather conditions during cereal-growing seasons. For both wheat and maize, seasons with wet weather, followed by an increased amount of precipitation, influence higher contamination frequency as well as a higher concentration of DON. Further, in years in which DON is detected with a significantly higher frequency and concentration than average, DON derivatives, mainly 3-acetyl deoxynivalenol (3-ADON) and 15-acetyl deoxynivalenol (15-ADON), as well as other trichothecenes, were also dominant among mycotoxins [6,55]. This situation was evidenced in Serbia, since in maize harvested in 2014, significantly higher concentrations of DON and other trichothecenes were detected, in comparison to other years from the last decade (2012–2022). DON contaminated 100% of examined maize samples, with the highest detected concentration reaching 16,350 µg/kg, while 3-ADON and 15-ADON were also detected with the high frequencies of 100% and 98% of the examined maize samples, respectively. In Serbia, the maize-growing season in 2014 was characterized by an extremely high amount of precipitation, while during certain months, the maximum recorded value of precipitation was registered since meteorological observations were established in Serbia [6,56,66]. The same situation was noticed in Croatia. Wet weather during the maize-growing season in 2014 resulted in increased contamination frequency as well as detected concentration of DON in comparison to other years from the last decade [66]. It is important to point out that several previous studies put Croatia among countries for which *Fusarium* contamination is considered characteristic [52,54,76,76,77,78]. A consistent warming trend, spanning from 0.2 °C per decade along the Adriatic up to 0.5 °C per decade in lowland Croatia, has been observed since the second half of the 20th century in Croatia. The increase in air temperature has resulted in a larger amount of accumulated heat as well as a prolongation of the growing season in Croatia. A weak precipitation trend has been observed on an annual level in Croatia, while the strongest negative precipitation trend along the Adriatic coast and its hinterland (−5–−15% per decade) was observed during the summer months [79]. Following this, it can be expected that aflatoxins would be prevalent in the future in the Croatian region, as well as in all Balkan regions.

Koletsi et al. [80] investigated the occurrence of mycotoxins in raw materials in Europe in the period 2012–2019 and also concluded that DON most frequently contaminates maize and wheat, while the level of contamination is strongly correlated with recorded weather conditions. The fact that DON is the most prevalent mycotoxin across Europe is also confirmed in multi-year surveys conducted by the Biomin company [75]. These World Mycotoxin Surveys represent one of the most comprehensive data sets on worldwide mycotoxin occurrence with a long tradition of around twenty years. These surveys apply modern analytical tools and include more than 15,000 samples of different agricultural commodities (corn, wheat, barley, finished feed, oats, etc.) per year and give detailed insights on the incidence and risk of the six major mycotoxins (AFs, OTA, ZEN, DON, FUM, and T-2). Multi-year surveys indicate that there is variation in mycotoxin occurrence between regions, indicating different risk levels. Among the regulated mycotoxins in Europe, DON is the most prevalent mycotoxin, with an average incidence higher than 50%, followed by two other *Fusarium* mycotoxins, FUMs and ZEN. Aflatoxins and OTA occurred with an average incidence of 10%, while their occurrence in Northern regions was lower compared to Southern regions, especially in years characterized by drought conditions. The more recent data confirmed that over 90% of samples were contaminated with more than 10 different mycotoxins and metabolites, while the average number of mycotoxins and metabolites per sample varied between 30 and 40. The most commonly detected fungal metabolites were moniliformin, bikaverin, aurofusarin, culmorin, enniatins, etc. Besides Biomin surveys, more and more recent studies indicate that some raw materials contain several dozens of different mycotoxins, which calls into question the synergistic effect since a mixture of different mycotoxins may generate additive or synergistic effects in humans and animals [81,82,83]. 

In accordance with the literature data published in recent years, the incidence of FUMs in maize was reported as high. A high level of FUMs contamination in maize is firmly linked to its growing conditions—the warmer and more humid the climate, the greater the likelihood of mycotoxin presence in the kernels. The predominant mycotoxin is FUM B1, and its high prevalence in maize has been noted in many countries all over the world. This is the reason why, from recent decades to the present day, there is a constant tendency to minimize their occurrence in maize [6,14,54,74].

## 5. Predictions for Certain Geographic Regions

In the last century, global temperature has risen by 0.7 °C, while in Europe the temperature rise was 1 °C. The hottest year ever seen in Europe was the year 2020 [14], while in the last 14 years, as many as seven hottest years have been registered. During the same time span, the amount of precipitation in northern Europe rose by 10–40%, while in southern Europe a drop in that amount was witnessed [22]. More recent notions point towards the fact that the coming decades are expected to bring a two- to three-fold rise in atmospheric carbon dioxide concentrations (i.e., from 350–400 to 800–1200 ppm), as well as a temperature rise of 2–5 °C and prolonged droughts [13,28,39,84,85,86]. Projections show that in the next century, the increase in global average annual temperature could be 1.4–5.8 °C, and in Europe 2.0–6.3 °C, and that cold winters will almost completely disappear by the end of the century, while warm summers will be even more frequent [14]. 

A particular “climate change strike” is expected to take place in southern Europe, consisting of elevated carbon dioxide atmospheric representation (doubled as compared to the average level registered in recent decades), temperature rise (by 2–4 °C), and extreme amounts of precipitation alternating with droughts. Predictive models demonstrate that northern Europe might even benefit from these changes, while the Mediterranean might be the focal point in which these changes will lead to numerous negative outcomes in terms of precipitation extremes, elevated temperatures, and elevated carbon dioxide levels. The maturation of cultivars in southern and central Europe might be premature compared to the current norm. In some of the south and southeast European countries (Portugal, Spain, south of France, Italy, Slovenia, Greece, Malta, Cyprus, Bulgaria, and southern Romania), a rise in temperature of 4–5 °C is foreseen, together with reduced water availability, especially during the summer months. In countries in western and Atlantic Europe (e.g., Benelux, western and northern France, northern Germany, the United Kingdom, the Republic of Ireland, the Netherlands, and Denmark), the temperature rise may span from 2.5 to 3.5 °C (2–3 °C in Great Britain and the Republic of Ireland), while the summers are anticipated to be drier and warmer [87]. 

Due to the increased amount of precipitation and its increased intensity, heavy storms and floods are foreseen to become more frequent, especially during the winter. In Norway, Sweden, Finland, and Baltic countries (of northern Europe), a temperature increase ranging from 3 to 4.5 °C is expected, together with an increase in annual precipitation amount of up to 40%, resulting in a high flood risk [88]. In central Europe (Poland, Czech Republic, Slovakia, Hungary, northern Romania, southern and eastern Germany, and eastern Austria), a temperature rise of 3–4 °C is forecasted (4–4.5 °C in the central European and Black Sea regions), while the amount of precipitation is expected to rise in winter and drop in summer, resulting in an increased flood risk [87]. Olesen et al. [89] investigated the impacts and adaptation of European crop production systems to climate change. This study indicates that the most negative effects were found for the continental climate in the Pannonian zone, which includes Hungary, Serbia, Bulgaria, and Romania. The authors predicted that this region will suffer from increased incidents of heat waves and droughts without the possibility of effectively shifting crop cultivation to other parts of the year.

It is fair to assume that climate change will have a huge general impact on aflatoxin crop contamination. More severe contamination with aflatoxigenic moulds will turn the most toxic representative of the aflatoxin group, AFB1, into a significant threat to food safety, primarily in eastern Europe, on the Balkan peninsula, and in the Mediterranean [20,21]. Modelling of potential climate change impact on the phenology of wheat grown in northwestern Europe has led to the expectation that winter wheat will both bloom and mature a week or two earlier than usual, while crop contamination with DON will become more common [27]. 

Many current predictions of the impact of climate change on mycotoxic moulds are based on historical or current climate conditions with an emphasis on the relationship between water availability and temperature [35]. In general, it is expected that climate change may increase the contamination of crops with mycotoxins; however, due to the complexity of the relationship between moulds, crops, and environmental factors, further research is necessary to confirm this thesis [27,86,90].

According to estimates, Australia will become far too hot, with a drastic temperature rise until 2100, and prolonged dry periods that will reduce water availability and make cultivation of a number of crops virtually impossible [87,91]. As for Asia, due to the lack of relevant data on this continent, mycotoxin contamination risk in the times to come will be more difficult to assess. Nevertheless, more pronounced flood-inflicted damages, a more intense mould penetration via damaged crops, and consequent rise in mycotoxin levels are definitely to be expected. However, in regions currently characterized by a hot climate, the environmental temperature may rise to an extreme level [24] capable of leading to the extinction of some mould species. For instance, the study conducted by Iqbal et al. [92], devoted to aflatoxin presence in chili, demonstrated that the temperature of 53.7 °C registered in Pakistan reduced the level of mould contamination, and hence also the level of aflatoxins.

The Latin-American scenario foresees significant temperature rises and a reduction in soil water content. Rainforest will turn into savannah-like grassland, and semi-dry soil cultures will be replaced by dry vegetation. Tropical species will perish in significant amounts and numbers, and crop productivity will drop [93]. In North America, floods and high temperatures will lead to storage-related problems in favour of mould growth and mycotoxin production. In regions currently characterized by a subtropical climate, such as the southern United States, the production of tropical cultures, such as coconut, maize, soy, coffee, and cocoa, may reach an optimum [94]. Elevated carbon dioxide atmospheric representation, temperature rise, and extreme amounts of precipitation exchanging with droughts are expected in parts of North and South America; for instance, in Brazil, as well as in Africa and Asia [87]. Operating under the assumption that tropical climate shall gradually become typical of subtropical regions, there is an expectation that developed countries will better cope with more severe mycotoxin contamination of tropical cultures than developing countries [87].

## 6. Further Research and Prevention Strategy

**Figure 2:**
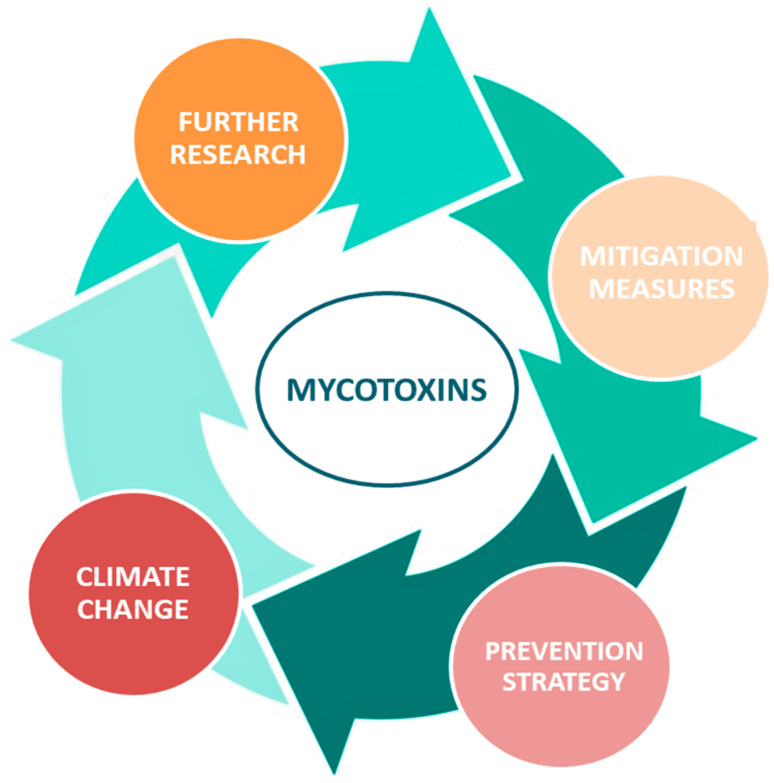
Necessary steps to prevent an increase of mycotoxin risk in a predicted climate change scenario.

Rapidly growing food and feed contamination face a great challenge from current as well as predicted climate change, and therefore there is a requirement for reinforced measures based on the multidisciplinary approach, including prevention strategy, mitigation measures, and further research (Figure 2). 

Prevention of mycotoxin contamination of food raw materials is generally considered to be more important than any containment “after the fact”. Therefore, for the prevention and significant reduction of mycotoxins in the whole agro-food chain, there is a need for the adaptation and implementation of the principles of good agriculture (GAP) and good manufacturing practices (GMP). For this purpose, several of the following principles of GAP may be implemented: usage of genotypes resistant or tolerant to toxigenic moulds, insects, and drought; biological control; irrigation; crop rotation; herbicide application; etc. Further, GMP is essential for reducing levels of mycotoxins in the food supply chain. Key parts of GMP include hazard analysis at critical control points (HACCP). HACCP implementation comprises a series of preventative procedures employed to the end of defining critical control points at which mycotoxigenic moulds and mycotoxins may enter the food chain, with the ultimate goal of ensuring food safety [95]. 

Which principles will be applied depends very much on the type of contaminated material as well as the present mycotoxin. For example, in cereals, some mycotoxins are usually considered pre-harvest problems (mainly *Fusarium*), while others (mainly *Aspergillus*) are recognized as post-harvest problems. However, some mycotoxins can be both a pre-harvest and post-harvest problem since they contaminate raw materials pre-harvest and require the application of certain post-harvest practices for handling and reducing mycotoxin contamination.

Given the concomitant appearance of mycotoxins in certain raw materials and consequently also in final products, notions on mycotoxin interactions are vital for the development of strategies aiming to prevent mycotoxin contamination and mycotoxicoses. Surveillance activities and contamination prevention should also be targeted at climate factors. Within this context, it is essential to fully understand the optimal temperature and relative humidity boundaries within which mould growth and mycotoxin production take place, especially when it comes to the major mould genera (*Aspergillus*, *Penicillium*, and *Fusarium*). Driven by these data, drying and storage practices exercised with both raw materials and final products should be designed so as to avoid toxicogenic mould colonisation and mycotoxin production [96]. 

At present, research employing “predictive modelling” to the end of identifying geographical regions in which the impact of interactive climate factors may result in the widest spread of toxigenic moulds and consequently the most severe mycotoxin contamination, is becoming ever more significant. The outcomes of these studies are linked to the food chain and the impact of interactive climate factors on food availability in the future. Of note, mycotoxins frequently occur concomitantly and have synergistic effects in their host organisms; in view of the foregoing, extensive research has been conducted in order to develop risk assessment models suitable for the assessment of multiple mycotoxin contamination cases [22].

Contamination minimisation strategies, linked to the knowledge of climate impact, should be based on a combination of several mitigation measures, including growing of fairly resilient cultivars, effective pre-harvest agricultural practices, application of chemical and biological anti-pest and anti-fungal treatments, and comprehensive insight into regional weather patterns. For a given crop, the planting strategy should be tailored based on knowledge of predominant climate characteristics, thereby taking local crop preferences and current regional and/or state production practices into account. Factors of the utmost importance for contamination control are crop rotation, agricultural techniques, variety or hybrid selection, and appropriate fungicide application [97]. 

Cereals should be dried in a manner allowing for the reduction of humidity level below that needed for mould growth during storage. If the water activity of the cereals is less than 0.65, this generally corresponds to a moisture level of 15 percent, which is relevant for preventing the growth of numerous types of moulds that can attack fresh crops. Even a tiny change in humidity level (that of <0.5%) may lead to mycotoxin contamination of foodstuffs in levels surpassing the maximal permissible ones [43]. Furthermore, appropriate handling procedures in terms of segregation, restoration, withdrawal, or redirection of use of cereals potentially dangerous to human and animal health should be implemented. During drying, any actions capable of damaging the grain should be avoided [96]. Shortening of the period during which wet, recently harvested crops are piled before drying and cleaning is vital for the reduction of mould growth risk. Maintenance of environmental hygiene in terms of pest and mould contamination prevention is a critical point both for medium- and long-term storage of goods, as well as for their transport, given that raw materials and final products circulating on the global market are transported over long distances and various climatic regions. Numerous foodstuffs are hygroscopic, i.e., prone to absorbing water, which allows for the growth of toxicogenic moulds and mycotoxin contamination. Therefore, food packaging should be clean and dry, hindering insect penetration and providing for the appropriate aeration of the storage environment, all s to the effect of maintaining an adequate and constant storage temperature [98]. 

Bearing climatic factors in mind, one should define the use of appropriate, duly registered, and duly approved insecticides and fungicides, or the use of alternative preservation techniques [97]. Chemicals used to the above end should be chosen with particular caution, so as to prevent any damage to the grain inflicted by their use. The choice should be guided by the intended grain end-use, while the duration of chemical treatment should be strictly limited. In order to make the control measures effective, it is vital to ensure the availability of relevant information on the eco-physiological impacts of abiotic and biotic stress on toxigenic moulds and their ability to produce mycotoxins [95]. This information is key to the better understanding and monitoring of the most prominent critical control points within the food production frame and allows for the optimisation of preventative strategies in each and every stage of the food chain. 

As for climate change, it is well known that burning of fossil fuels exercised by various industries leads to carbon dioxide emission; the removal of this gas is reduced due to the continual decrease of forest areas, which are its main consumers [99]. Increased atmospheric concentrations of this greenhouse gas lead to increased heat absorption, which, in turn, leads to changes in air temperature, precipitation amounts, and other climatological elements. In view of these facts, mycotoxin contamination prevention should be put in a broader context of global efforts to downsize carbon dioxide production and environmental contamination in general. 

Further research worldwide should be focused on the impact of numerous environmental variables, including temperature, pest attacks, and nutrients’ availability on mycotoxin production and presence in food. They should be targeted at the influence of various climate scenarios, thereby taking the interaction between elevated carbon dioxide levels, environmental temperature, and drought-induced stress into account. Although several models within the frame of climate change linked to the prevalence of certain mycotoxins [100,101,102,103,104] have already been compiled, research targeted at the possible prevalence of more mycotoxins in more cereal types is still pending. Compilation of well-founded models dealing with climate change consequences would enable both producers and consumers to predict future mould prevalence and mycotoxin contamination. Given the fact that climate change witnessed today goes beyond boundaries that can be solely attributed to natural variability, to foresee their impact it is almost impossible to assess human responsibility in this regard. However, air quality improvement, on which human factor definitely has a bearing, could without doubt strengthen global efforts to alleviate the consequences of ever more intense climate change. 

Four major research areas expected to provide useful data on the future influence of climate change on mycotoxin contamination risk have been identified, including: (i) the use of available simulations of future climate scenarios on a regional scale; (ii) the use of existent data and the performance of additional research on possible future influences of climate factors on the prevalence of various mould species and mycotoxin concentrations; (iii) development of models capable of predicting the levels of most significant mycotoxins, such as AFs, FUM, and DON, primarily in maize, but in other cereals as well, thereby taking future climate scenarios into due account; and (iv) performance of research on the impact of climate change on food safety in foreseeable and distant future, focusing thereby on mycotoxin-related economic and health risks [22,105].

## 7. Conclusions

It has already been well documented that food and feed contamination incurs huge costs worldwide, which will most probably rise due to the increased frequency and intensity of climate extremes. Climate change produces a confluence of factors that can act together and alter attributes of the fungus, the environment, and the host, which can then drive the emergence of novel, uncommon, or adapted fungal species, with consequences for health, biodiversity, and food and feed security. In addition, the aflatoxin risk in maize will be likely higher as a result of global warming, and it has been predicted that in Europe in the next 50–100 years, *A. flavus* and aflatoxins will become of the greatest concern. Additionally, the representation of certain mycotoxigenic *Fusarium* species is changing ever more substantially, while the frequency of contamination with *F. graminearum*, as a species capable of producing several toxic mycotoxins, is increasingly observed in northern and central Europe. Thus, as the first approach to control the development and growth of toxigenic filamentous fungi, and thus also their metabolites, i.e., mycotoxins in food and feed, the use of good agricultural practices and good manufacturing practices in the field and during the handling, storage, processing, transportation, and distribution is strongly recommended. Since environmental conditions are unpredictable factors that influence mycotoxin occurrence in food and feed, an interdisciplinary approach is essential to solving the threat caused by climate change. On the other, postharvest decontaminating approaches can be a last resort to solve the issues with mycotoxins in food and feed.

## Figures and Tables

**Table 1:** Overview of climate change in the world [24].

Continent	Warming	Precipitation
Africa	Increased mean air temperatures; increased annual number of hot days and warm nights; heatwaves became longer and more intensive	Aridity increase due to decrease in precipitation in North Africa; increased trend of heavy rainfall followed by more frequent droughts in West Africa; more frequent and prolonged droughts in East and Southern Africa; increased number and intensity of extreme precipitation events in Southern Africa
Asia	Increased mean air temperatures followed by increased number of hot days and warm nights throughout continent; more frequent and longer heatwaves in South and East Asia	Observed annual precipitation trends are different depending on region; decreased precipitation and increased evapotranspiration are observed in West and Central Asia; increase in heavy precipitation in South, Southeast, and East Asia
Australia	Increased mean air temperature; heatwaves have increased in frequency and duration in most regions; increased number of extremely hot days	Increase in rainfall over northern Australia; decreased rainfall in southwestern and southeastern Australia in the period April–October; extreme rainfall intensities increased in many locations; less frequent droughts in northern and central Australia; more frequent droughts in southwest
Europe	Increased mean and maximum air temperatures in all regions followed by more frequent and prolonged heatwaves; increased occurrence of hot days and warm nights; cold extremes have decreased	Mean precipitation increased over Northern, Eastern, Western, and Central Europe; precipitation extremes increased in Northern and Eastern Europe; decreasing trend of precipitation observed in Mediterranean; more frequent droughts in Mediterranean; less frequent droughts in Northern Europe
Central and South America	Increase in the frequency and intensity of warm extremes and heatwave duration; increased mean and maximum temperatures; decrease in the intensity and frequency of cold extremes	Decreasing trend in precipitation in Central America; intensity and frequency of droughts have increased in many parts of Southern America; increasing trend of precipitation was observed in southeastern part of South America
North America	Increased mean and maximum air temperatures; increased occurrence of hot extreme events	Annual precipitation has increased in northern and eastern parts and decreased in western parts; increased frequency and intensity of heavy precipitation events in USA

## Data Availability

The datasets generated for this study are available on request to the corresponding author.
